# Analysis of the Associations of Measurements of Body Composition and Inflammatory Factors with Cardiovascular Disease and Its Comorbidities in a Community-Based Study

**DOI:** 10.3390/biomedicines12051066

**Published:** 2024-05-11

**Authors:** Nader Tarabeih, Alexander Kalinkovich, Shai Ashkenazi, Stacey S. Cherny, Adel Shalata, Gregory Livshits

**Affiliations:** 1 Department of Morphological Sciences, Adelson School of Medicine, Ariel University, Ariel 40700, Israel; nadertar@gmail.com (N.T.); shaias@ariel.ac.il (S.A.); 2 Department of Anatomy and Anthropology, Faculty of Medicine, Tel-Aviv University, Tel-Aviv 69978, Israel; alexander.kalinkovich@gmail.com (A.K.); stacey.cherny@gmail.com (S.S.C.); 3 The Simon Winter Institute for Human Genetics, Bnai Zion Medical Center, The Ruth and Bruce Rappaport Faculty of Medicine, Technion, Haifa 32000, Israel; adel.shalata@gmail.com

**Keywords:** hypertension, hyperlipidemia, type 2 diabetes mellitus, adipokines, GDF-15, body composition, inflammation, multivariable statistical analyses

## Abstract

The associations of cardiovascular disease (CVD) with comorbidities and biochemical and body composition measurements are repeatedly described but have not been studied simultaneously. In the present cross-sectional study, information on CVD and comorbidities [type 2 diabetes mellitus (T2DM), hypertension (HTN), and hyperlipidemia (HDL)], body composition, levels of soluble markers, and other measures were collected from 1079 individuals. When we examined the association of each comorbidity and CVD, controlling for other comorbidities, we observed a clear pattern of the comorbidity-related specific associations with tested covariates. For example, T2DM was significantly associated with GDF-15 levels and the leptin/adiponectin (L/A) ratio independently of two other comorbidities; HTN, similarly, was independently associated with extracellular water (ECW) levels, L/A ratio, and age; and HDL was independently related to age only. CVD showed very strong independent associations with each of the comorbidities, being associated most strongly with HTN (OR = 10.89, 6.46–18.38) but also with HDL (2.49, 1.43–4.33) and T2DM (1.93, 1.12–3.33). An additive Bayesian network analysis suggests that all three comorbidities, particularly HTN, GDF-15 levels, and ECW content, likely have a main role in the risk of CVD development. Other factors, L/A ratio, lymphocyte count, and the systemic inflammation response index, are likely indirectly related to CVD, acting through the comorbidities and ECW.

## 1. Introduction

Cardiovascular diseases (CVDs) encompass various disorders, including coronary artery disease (CAD), myocardial infarction (MI), angina pectoris, congestive heart failure (CHF), and rheumatic heart disease [1,2,3]. Cardiovascular disease (CVD) is a leading cause of morbidity and mortality worldwide [4]. The development of CVD is often associated with various comorbidities, including obesity, type 2 diabetes mellitus (T2DM) [5,6,7], hyperlipidemia (HLD) [8,9], and hypertension (HTN) [10], which are all well-known risk factors for CVD. The major underlying pathological mechanism of CVD is atherosclerosis, characterized by a complex interaction between inflammatory and metabolic factors [11]. Its causal relationship with the aforementioned comorbidities T2DM, HLD, and HTN is also well established [12,13,14,15]. This creates a solid basis for their analysis in combination with CVD.

However, recent data suggest that several newly recognized variables play a role in the pathogenesis of CVD and its comorbidities, with these variables being potentially related to or even accelerating the development of atherosclerosis. They include changes in body composition, which are also associated with the risk of CVD [16]. For example, individuals with low fat mass (FM) and high skeletal muscle mass (SMM) have a significantly lower CVD mortality risk in comparison with overweight and obese individuals [17]. It is also known that adipokines, specifically leptin and adiponectin, are involved in the pathogenesis of obesity-associated CVD [18,19]. However, their effect on regulating cardiovascular function remains controversial. Low adiponectin and elevated leptin levels, separately, are associated with severe CVD [20], but in most cases, high circulating leptin and adiponectin levels do not show any beneficial effects [18]. In this regard, the leptin/adiponectin (L/A) ratio was shown to be a stronger predictor of the risk of CAD compared to leptin or adiponectin serum level alone [21,22]. Another adipokine of potential interest is chemerin, which is involved in vascular inflammation, angiogenesis, and blood pressure modulation [23]. The latter study suggests that chemerin potentially plays an important role in the pathogenesis of CVD and proposes perspectives for developing chemerin-targeting therapeutic agents for the treatment of CVD.

Recent publications point to the significant and consistent association of growth and differentiation factor 15 (GDF-15) with several inflammation-mediated conditions and metabolic diseases, including T2DM, obesity, and HTN [24,25], and to the increased risk of CVD [26,27], in particular atrial fibrillation, CAD, MI, and cardioembolic stroke [28,29,30]. GDF-15 belongs to the multifunctional transforming growth factor-β (TGF-β) superfamily of proteins [31,32], However, the mechanisms underlying GDF-15 involvement in the above conditions and CVD remain poorly understood. Though less extensively studied, plasma levels of a hepatokine called follistatin have also been linked to several metabolic conditions [33] and associated with an increased risk of mortality and heart failure in CVD patients [34].

There is a growing body of evidence suggesting that inflammation is a crucial mechanism underlying the development of various diseases, including CVD [35]. The systemic inflammation response index (SIRI) is a novel prognostic marker based on the composition ratio of peripheral blood neutrophil, monocyte, and lymphocyte counts [36]. Elevated levels of SIRI have been linked to cancer, rheumatoid arthritis, and acute ischemic stroke [37,38,39].

Altogether, the previous studies suggest the contribution of body composition parameters, inflammation, adipokines, and GDF-15 levels in CVD pathogenesis, although the precise relationships among these variables have not yet been fully elucidated. Hence, the major aim of the present study was to comprehensively evaluate the extent to which CVD is associated with the combined effect of these factors, controlling for age and sex, and considering metabolic comorbidities (T2DM, HLD, and HTN) as intermediate conditions. We attempted to uncover the possible causal network underlying the relationships among these variables and CVD by evaluating all the variables in a well-defined and well-studied population.

## 2. Materials and Methods

### 2.1. Study Population Design and Ethics

This study was a case–control, community-based, cross-sectional study. The data were collected from 1079 individuals (mean age 43.0 ± 13.8 years) enrolled in outpatient clinics in the small city of Sakhnin (Israel) from 2015 to 2022. All participants were from the ethnically and culturally homogeneous population of Israeli Arabs, comprising 98 nuclear and more complex three-generation families [40,41]. They provided complete medical histories and consented to provide access to their medical records. The inclusion criterion for the study group was an age of 18 to 78 years. The exclusion criteria were pregnancy, traumatic disorders, systemic inflammatory or autoimmune disorders, neoplastic disease, and a history of malignancy. Certified and experienced nurses assessed all participants in the study population. Demographic data, anthropometrics, body composition measurements, comorbidities, history of CVD, and blood samples (30 mL) were collected from all individuals in the study population. Blood samples were used to assay plasma concentrations of biochemical factors relevant to the present study.

This research was approved by the IRB-Helsinki Committee (Number: 042/2013K, Date: 4 November 2013) of the Meir Medical Center, Kfar Saba, Israel, and the Ethics Committee of Tel Aviv University, Tel Aviv, Israel. Written informed consent was obtained from all participants before their inclusion.

### 2.2. Definition of CVD and Comorbidities

A detailed medical history was obtained by two methods. First, during interviews, the subjects were asked to report the medical conditions they had and received treatment for between 2015 and 2021, and second, their medical records were checked for the diagnoses of CVD and comorbidities including HTN, HLD, and T2DM. CVD in this study was defined according to the WHO (2021) criteria and included CHD, CHF, MI, and angina pectoris. CVD diagnosis was determined according to a cardiologist as suffering from CVD, and those hospitalized for heart failure comprised the affected group.

The control group was defined as the remaining individuals who did not suffer from the CVD conditions or any of the comorbidities examined in this study: HTN, HLD, and T2DM. The corresponding sample sizes are given in Table 1.

### 2.3. Demographic, Anthropometric, and Body Composition Assessment

Demographic, anthropometric, and body composition data were collected from the study population and recently described in detail [40]. They included height (cm), weight (kg), waist and hip circumferences (cm), calculated body mass index (BMI) in kg/m^2^, and waist-to-hip ratio (WHR) in mm/mm. Body composition parameters were assessed by bioimpedance analysis (BIA) using the BIA101 device (Akern Bioresearch, Pisa, Italy), a safe, reliable, accurate, and inexpensive method, as previously described [42,43]. BIA gives several body composition-associated measures, of which we included the evaluation of fat mass (FM) and skeletal muscle mass (SMM) in kilograms and total body water (TBW) and extracellular water (ECW) in liters. TBW and ECW were chosen due to their fundamental physiological significance [44], in particular because they may serve as indicators of adiposity and inflammation [45]. Body mass components were used as ratios to body weight, such as FM/WT and SMM/WT, as they are interrelated and dependent on body weight.

### 2.4. Measurement of Soluble Biomarkers

Venous blood samples were collected from all study individuals after an overnight fast. They were centrifuged for 15 min at 1800× *g* at 4 °C within one hour of collection. Plasma fractions were separated and stored in aliquots at −80 °C. The levels of soluble markers were determined by ELISA using the DuoSet kits (R&D Systems, Minneapolis, MN, USA) according to the manufacturer’s protocols. The detection limits were as follows: 7.8 pg/mL for GDF-15, 46.9 pg/mL for follistatin, 16.7 pg/mL for chemerin, 31.2 pg/mL for leptin, and 62.5 µg/mL for adiponectin. The intra- and inter-assay coefficients of variation were between 2.3 and 8.6%. In addition, blood assaying of high-sensitivity CRP (hs-CRP) levels and prothrombin time (PT) was carried out. Before statistical analysis, the original measurements of the biomarkers deviating from the normal distribution assumptions were log-transformed.

### 2.5. Inflammatory Biomarkers

These biomarkers included total lymphocyte, monocyte, neutrophil, and platelet counts. Using them, the systemic inflammation response index (SIRI) was calculated by using the following formula: (neutrophils × monocytes)/lymphocytes [39]. Platelets are well-known blood clotting factors, with substantial emerging data suggesting that they may play considerable roles in immune responses and inflammation [46].

### 2.6. Statistical Analysis

The statistical analysis included three main stages. In the first stage, we aimed to identify the major covariates (potential predictors) for CVD and the comorbidities (HTN, HLD, and T2DM). Continuous variables were compared between the affected and non-affected (control) groups using *t*-tests and parametric and non-parametric (Kruskal–Wallis) ANOVAs, followed by correlation/regression analysis. These analyses were conducted using Statistica 64 (TIBCO Software, Version 13.5) and R [47].

Next, we tested the independent relative effect (association) of each of the covariates detected above on each of the comorbidities and CVD status. The results of the analyses were compared. To this aim, we implemented logistic mixed-effects models with the *relmatGlmer* function package for binary dependent variables from the R package lme4qtl [48], which in addition to the simultaneous testing of the association between covariates and dependent variables, also account for familial composition by use of the kinship2 package [49] for R to generate kinship matrices. Missing data were imputed using the R package mice [50] with the default options prior to analysis.

At the final stage of analysis, we included variables with significant associations obtained in previous stages in an additive Bayesian network (ABN) analysis to explore the possible underlying causal structure of the variables examined.

### 2.7. Additive Bayesian Network (ABN) Modeling

To explore the possible underlying causal structure for the variables examined, we used ABN models [51], as implemented in the R package ABN, version 3.0.1 [52,53], with JAGS software, version 4.3.0, to perform a parametric bootstrap and correct for overfitting [54]. While ABN modeling does not require any causal assumptions, if there are strong theoretical reasons for making assumptions, they should be made, and they will aid in finding the best model. We therefore did not permit causal arcs that were theoretically nonsensical. Our restriction was that age at testing could not be caused by any other variable and that CVD was the end event. Before analysis, we imputed missing data, including only the variables used in the ABN model, using the R package mice [50] with the default options, since ABN requires complete data for analysis. We used a four-stage analysis pipeline to arrive at a final causal model that guards against overfitting, as previously described [55,56]. Because it is theoretically possible that distinct causal structures could produce the same likelihood of the data [57,58], we caution that there are equivalent models with the causal direction reversed, though this is not of major concern due to the strong theoretical basis for the direction of some of the arcs.

## 3. Results

### 3.1. Characteristics of the Study Population

Table S1 (electronic Supplementary Materials) presents the mean values of the variables in the study population, separated by sex. The sample size consisted of 490 men and 589 women, with no significant differences in age between the groups (42.76 ± 0.62 years vs. 43.20 ± 0.56 years, *p* > 0.05). The prevalence of CVD also showed no significant difference between women and men (9% [52/589] vs. 12% [59/490], *p* > 0.05). Women had significantly higher body composition variables related to adipose tissue mass (BMI, FM/WT, and ECW) than men, while men had higher waist circumference, SMM/WT, and TBW values. The lymphocyte counts and SIRI levels were significantly higher in men than in women (SIRI: 0.87 ± 0.02 vs. 0.70 ± 0.01, *p* < 0.0001), yet there was no significant difference in CRP levels. The levels of GDF-15 (pg/mL) were significantly higher in men compared to women (520.47 ± 14.69 vs. 460.73 ± 13.10, respectively, *p* = 0.002), while the circulating levels of leptin and adiponectin, as well as the L/A ratios, were higher in women. No differences were found between men the and women concerning the other variables. The anthropometric measurements and body composition were significantly intercorrelated in both sexes, as shown in Table S2. To avoid redundancy and collinearity in further analyses, only variables with significant correlations with comorbidity categories and CVD status were selected.

### 3.2. Associations of Covariates with CVD and Comorbidities

A series of univariate analyses of the associations between covariates and CVD and comorbidities are presented in Table 1. At this stage of the analysis, there was an overlap between the comorbidities, i.e., there were individuals diagnosed with two or more diseases. As seen, individuals with HTN, HLD, T2DM, and CVD tend to be older and exhibit higher obesity measures (BMI, waist circumference, WHR, and FM/WT) than those without any comorbidity or CVD, even after controlling for sex and age differences. The levels of ECW content in all affected groups were significantly higher in comparison with the control group, while the SMM/WT measurements were significantly lower in patients with CVD. The plasma levels of GDF-15, chemerin, and follistatin; L/A ratios; and the lymphocyte counts were significantly higher in the patients with comorbidities and CVD compared to the controls independent of age and sex differences. Notably, when comparing the PT, CRP, and SIRI levels, we found that they were significantly higher only in individuals with CVD compared to healthy individuals.

### 3.3. Multivariable Analysis

At this stage, all potential predictor variables (covariates) that were significantly associated with comorbidities and CVD status in the univariate context were analyzed by mixed-effects logistic regression models to examine the combined associations of the body composition measurements and plasma levels of soluble markers, controlling simultaneously for familial relations in the sample. These models take into account also the effect of the complementary comorbidity on the comorbidity in test. In the CVD analysis, all three comorbidities were included in the regression analysis as covariates. The results are summarized in Table 2 and Table 3. Other parameters tested, which were significantly elevated in patients with comorbidities and CVD compared with the control group (Table 1), were not retained in the final regression equation as independently associated covariates.

The analysis was conducted in two stages. First, the covariates’ associations with each of the three comorbidity categories (HTN, HLD, and T2DM) as dependent variables were examined. Age and sex were included in each analysis (Table 2). Next, all the retained significant covariates in the univariate analysis (Table 1) were tested with CVD status (Table 3, stage 1).

The analysis revealed a clear pattern of the comorbidity-related specificity of the association with the covariates (Table 2). HTN demonstrated highly significant associations with ECW, the L/A ratio, and T2DM, with corresponding *p*-values ranging between 0.01 and 5.31 × 10^−5^. HTN was a strongly age-dependent condition (*p* = 2.12 × 10^−10^) and correlated significantly (*p* = 4.21 × 10^−7^) with HLD. When HLD was examined, it also displayed a strong association with age, but the other associations provided in Table 1 were attributable to its highly significant associations with the two other comorbidities. The analysis of T2DM showed its highly significant associations with GDF-15 (*p* = 0.000001) and HLD (*p* = 1.09 × 10^−12^). T2DM showed no independent significant association with age but was moderately significantly associated with the L/A ratio and HTN.

Next, we conducted multiple logistic regression analyses with CVD status as the dependent variable to investigate the independent and combined effects of the covariates identified in the univariate context (Table 1) and, at the final stage, included the three comorbidities in the analysis as covariates. The results of the first stage showed that ECW, plasma GDF-15 levels, and all the included inflammatory indices showed independent and statistically significant associations with CVD (Table 3, stage 1). The calculated odds ratio (OR) ranged from 1.49 (1.19–1.87) for SIRI levels to 2.42 (1.78–3.30) for GDF-15. The overall significance of the model (vs. the model with no predictor variables), as assessed by the likelihood ratio vs. zero model, was also very high. Interestingly, the obesity variables (BMI, WHR, and waist circumference) showed no independent association with CVD. When the comorbidities were included in the analysis, ECW and GDF-15 levels remained significant, in addition to the comorbidities, whereas the L/A ratio and SIRI were no longer significantly associated with CVD (Table 3, stage 2).

### 3.4. Additive Bayesian Network (ABN) Analysis

**Figure 1:**
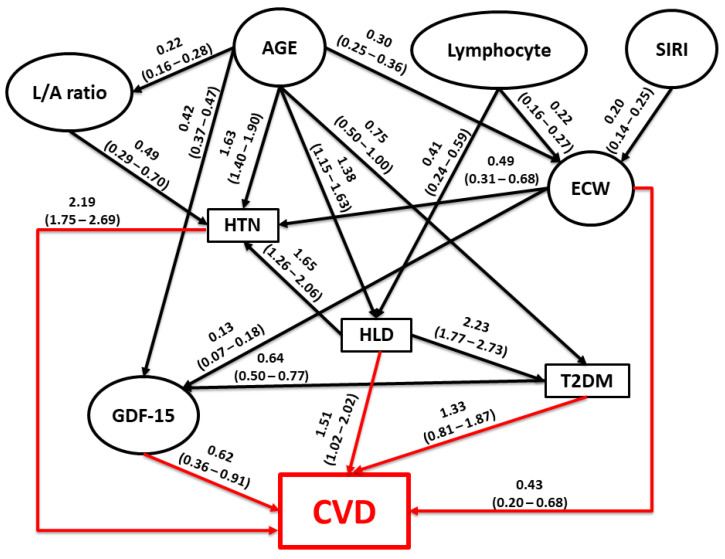
Directed acyclic graph among study measures, generated by additive Bayesian network modeling. Continuous variables are represented by ovals, and the squares represent binary variables. All quantitative variables were standardized before analysis. The coefficients on the arcs (paths) are the modes (beta) obtained from the posterior distributions of the coefficients, with the corresponding 95% credible intervals presented below in parentheses. The red arrows denote direct influences on CVD, and the black arrows indicate direct influences of CVD on other variables. Abbreviations: HTN, hypertension; HLD, hyperlipidemia; T2DM, type 2 diabetes mellitus; CVD, cardiovascular disease; ECW, extracellular water; L/A ratio, leptin/adiponectin ratio; GDF-15, growth differentiation factor-15; SIRI, systemic inflammation response index.

The relationships uncovered among the variables can be seen in Figure 1, with the parameter estimates shown on the arcs and 95% credible intervals in brackets under them. The procedure standardizes continuous variables before analysis. Most covariates in the study were significantly dependent on age except for lymphocyte count, SIRI, and CVD. The clinically most important links were the direct and independent connections found between each of the comorbidity categories (HTN, HLD, and T2DM) and CVD status (all consistently positive). Two inflammation-related factors, ECW and GDF-15, also demonstrated significant and presumably direct associations with CVD. Interestingly, the effects of other inflammatory factors, namely, lymphocyte count, SIRI, and L/A, that were significantly associated with CVD in the regression analysis, appear to be indirectly linked through the comorbidities (HTN, HLD, ECW, and GDF-15). It appears that T2DM affected CVD both directly and indirectly through GDF-15 levels, while by itself, it depended only on age and HLD.

## 4. Discussion

CVD is a leading cause of morbidity, disability, and mortality worldwide [2]. Given the significant impact of CVD on public health, continued research is needed to improve prevention, diagnosis, and treatment strategies to reduce its burden. In our study of ethnically homogenous 1079 individuals, we examined a range of factors whose potential involvement in the pathogenesis of CVD was previously reported but have not been evaluated together in a single study. The measured factors included CVD comorbidities (T2DM, HTN, and HDL), body composition parameters (BMI, waist circumferences, WHR, ECW, and FM/WT), and a range of circulating factors that are associated with inflammation and adipose tissue functions.

The most remarkable result observed at the first stage of the study was the clear pattern of the specific comorbidity-related associations with all the other studied variables when, in the regression analysis of each comorbidity (e.g., T2DM), we controlled for two others. For example, GDF-15, which showed highly significant and consistent associations with all three comorbidities and CVD in our univariate analyses, remained significantly associated with T2DM and CVD only, after adjustment for other covariates.

This makes the results concerning GDF-15 especially interesting. As mentioned in the Introduction, elevated circulating levels of GDF-15 are considered a relevant clinical biomarker for CVD [29,59,60], being linked to both cardiovascular and all-cause mortality [60,61]. However, reports suggest that GDF-15 is both protective [62,63] and a risk factor of CVD [64,65]. Some data suggest its potential role as a dynamic marker reflecting the course of CVD [65]. In our multivariable analyses, when we statistically controlled for the effect of the supplementary comorbidities on the comorbidity or CVD, we found that GDF-15 levels were independently associated only with T2DM [OR 1.87 (1.44–2.42)] and CVD [1.85 (1.39–2.46)]. This suggests that the association is related to some specific metabolic pathway characteristic of these pathological conditions. The involvement of GDF-15 in the pathogenesis of T2DM is well known [26,66,67], whereas reports on its association with HTN and HLD are controversial [24,25]. GDF-15 has been shown to modulate energy balance and glucose homeostasis, and its administration leads to promising beneficial effects against obesity and associated metabolic diseases in pre-clinical models [68]. Furthermore, the endogenous upregulation of GDF-15 is associated with resistance to diet-induced obesity, improved glucose homeostasis, and increased insulin sensitivity [69]. In a recent study, GDF-15 was shown to protect insulin-producing beta cells against pro-inflammatory cytokines and metabolic stress [70].

Although the elevation of circulating GDF-15 levels in various age-associated disorders, including CVD and its comorbidities, is well established, the mechanisms remain not fully understood. One possible mechanism is the activation of tumor suppression protein p53, which has been proposed to promote inflammation and insulin resistance in adipose tissue in both mice and humans [71]. Moreover, in vitro studies have shown that the upregulation of GDF-15 expression occurs in a p53-dependent manner [72], which, in turn, triggers the AMP-activated protein kinase (AMPK)–p53 signaling pathway [73]. Another proposed mechanism is mitochondrial dysfunction [69] as circulating GDF-15 levels are considered a reliable diagnostic marker for mitochondrial diseases [74,75,76]. Mitochondrial dysfunction, in turn, is closely associated with aging and is a major cause of many age-related diseases [77].

In our study, we also observed well-known associations for all three examined comorbidities with CVD. Based on the Akaike information criterion, model 2, including comorbidities as covariates, fits the data better than model 1, not including them into the analysis (Table 3). However, the extent of the associations and therefore the potential risk for CVD manifestation differed substantially. Thus, the OR estimated for HTN was 10.89 (6.46–18.38), vs. the much smaller, though still impressive, ORs of 2.49 (1.43–4.33) and 1.93 (1.12–3.33) for HLD and T2DM, respectively. All three comorbidities are metabolically attributable risk factors for CVD burden and mortality [78,79,80] closely associated with body composition changes [81,82] and inflammation [83,84]. HTN was repeatedly reported as one of the strongest risk factors for almost all CVD manifestations, including coronary disease, left ventricular hypertrophy and valvular heart diseases, and cardiac arrhythmias including atrial fibrillation, cerebral stroke, and renal failure [85,86]. It has been estimated that about 47% of CAD worldwide is attributable to HTN [87]. A significant association of CVD with HLD and T2DM is also well established [88,89,90,91], suggesting the reproducibility and reliability of our findings.

To the best of our knowledge, our study examined, for the first time, the independent associations in the same sample of CVD individuals, simultaneously controlling for the effect of each other, and additional covariates such as age and sex. CVD is an age-dependent multifactorial condition. As mentioned in the Introduction, body composition, in particular obesity, low muscle mass, and inflammatory factors, also play important roles in CVD pathogenesis. Yet, our final analyses (Table 3) showed that these factors became statistically insignificant after controlling for comorbidity effects and, thus, suggested that an age-dependent increase in CVD and the effects of obesity and inflammation are indirect and transformed via these covariates and ECW.

Remarkably, ECW survived all the adjustments and remained significantly associated with CVD (Table 3). The association of ECW with CVD is well explainable regarding the pathophysiology of CVD. ECW is significantly correlated with all the measures of body composition (Table S2, Supplementary Materials) and probably replaces them in final regression analysis. It is known that inflammation is accompanied by an increased blood supply to the damaged area, which, in turn, leads to an increase in local ECW. It therefore may serve as an indirect marker of inflammation and obesity [92]. On the other hand, studies have reported elevated ECW levels in individuals with left ventricular hypertrophy [93] and coronary artery calcification [94]. Elevated ECW content may aggravate endothelial and vascular dysfunction, which promotes atherosclerosis, leading to increased efferent pressure, which causes HTN [93,95], and elevated ECW content was also observed in our multivariable regression analysis (Table 2).

To better understand the complex inter-relationships between all these factors with CVD, we applied ABN modeling. As shown in Figure 1, GDF-15 levels, ECW content, and comorbidities (T2DM, HTN, and HDL) presumably have a main role in the risk of CVD development, although the strength of the association differ substantially. Regression estimates suggest that comorbidities, particularly HTN, are the main risk factors for CVD. Other factors, namely, the L/A ratio, lymphocyte count, and SIRI, are likely indirectly related to CVD, mediating their effects through the intermediate comorbidity phenotypes and ECW. Finally, GDF-15 has an interesting effect: according to the model, it is age-dependent and directly affected by ECW and T2DM. These associations are well interpretable considering GDF-15’s role as a marker of aging and anti-inflammation function [96]. Its direct effect on CVD, as seen on the DAG, is controversial, and the causal direction may be opposite, as arrows can often be reversed in ABN models and result in the same model fit. This is one of the limitations of the ABN method, and therefore, this relationship requires further confirmation.

Our study has several limitations. The most notable one is its cross-sectional design, which does not allow conclusions to be drawn about the causality of the associations found. Longitudinal studies are needed to establish causal relationships among CVD, comorbidities, and other measured factors and to evaluate their prognostic nature. Another limitation is that this study was conducted on a single ethnically and culturally homogeneous population, and there is potential for residual confounding that we unfortunately cannot address. For example, lifestyle variables such as stress, smoking, physical activity, food intake, and occupation were not collected in the present study. Therefore, additional studies in other populations are needed to generalize the findings reported in this study.

## 5. Conclusions

This study reports several novel findings with prognostic potential for CVD. To the best of our knowledge, this is the first study in which the diverse potential risk factors for CVD, including the comorbidities, characteristics of body composition, and circulating factors associated with adipose tissue functions and inflammation, were examined together in a defined population. In addition, we uncovered the possible direct (causal) and indirect relationships underlying the complex network of variables potentially affecting the risk of CVD. The important and novel results from our analysis suggest the association of specific comorbidities with risk factors.

The comprehensive associations found are complex and probably hierarchical, as illustrated by the ABN analysis. The present study assumes that some of the factors (in particular, comorbidities and especially HTN) are more likely to have a major and direct role in CVD pathogenesis than others. Some of the well-known risk factors, such as obesity and inflammatory factors, probably affect CVD indirectly through the intermediate health conditions. These observations, if confirmed, may contribute to a better understanding of the multifactorial pathogenesis of CVD and thus may lead to improved diagnosis, monitoring, prognosis, prevention, and treatment strategies for patients with CVD.

## Figures and Tables

**Table 1:** Comparison of the assessed variables between the control group and individuals affected with comorbidities and CVD.

Group Variable	Control (N = 706)	(1) HTN (N = 264)	(2) HLD (N = 343)	(3) T2DM (N = 181)	(4) CVD (N = 111)	P1	P2	P3	P4
Age (years)	38.69 ± 0.41	56.19 ± 0.67	54.23 ± 0.58	55.38 ± 0.75	56.33 ± 1.20	-	-	-	-
BMI (kg/m^2^)	27.78 ± 0.16	31.42 ± 0.30	30.83 ± 0.27	31.69 ± 0.36	31.73 ± 0.58	4.86 × 10^−7^	0.000005	0.000001	0.000002
Waist circumference (cm)	93.86 ± 0.41	104.48 ± 0.71	102.83 ± 0.65	105.15 ± 0.88	104.34 ± 1.22	9.27 × 10^−8^	0.000005	2.45 × 10^−8^	1.61 × 10^−7^
WHR	0.88 ± 0.002	0.95 ± 0.004	0.94 ± 0.004	0.96 ± 0.006	0.95 ± 0.007	0.0001	0.005	8.04×10^−9^	0.01
FM/WT (kg/kg)	0.31 ± 0.002	0.35 ± 0.005	0.34 ± 0.005	0.35 ± 0.006	0.34 ± 0.009	0.002	0.01	0.01	0.0001
SMM/WT (kg/kg)	0.33 ± 0.002	0.29 ± 0.004	0.29 ± 0.003	0.29 ± 0.004	0.29 ± 0.006	NS	NS	NS	0.01
TBW (L)	38.55 ± 0.24	40.88 ± 0.50	40.23 ± 0.41	40.93 ± 0.58	41.45 ± 0.72	9.77 × 10^−8^	0.004	4.84 × 10^−7^	0.001
ECW (L)	17.60 ± 0.11	20.35 ± 0.24	19.77 ± 0.19	20.12 ± 0.27	20.72 ± 0.33	1.06 × 10^−9^	0.005	0.000002	1.63 × 10^−8^
GDF-15 (pg/mL)	401.32 ± 7.54	658.57 ± 21.79	695.42 ± 26.11	785.12 ± 34.85	802.67 ± 46.26	0.000002	0.000007	0.001	4.16 × 10^−13^
Chemerin (ng/mL)	87.91 ± 0.86	103.69 ± 1.92	100.61 ± 1.60	103.36 ± 2.34	104.54 ± 3.15	0.00003	0.002	0.001	0.00001
Follistatin (pg/mL)	549.46 ± 16.35	700.99 ± 26.30	679.67 ± 22.51	732.21 ± 33.73	695.70 ± 36.10	0.003	0.001	0.001	0.04
L/A ratio	5.19 ± 0.20	8.83 ± 0.44	8.14 ± 0.37	8.75 ± 0.52	8.26 ± 0.74	0.0002	0.001	0.0002	0.00007
Lymphocyte (×10^9^/L)	2.13 ± 0.02	2.33 ± 0.04	2.35 ± 0.04	2.39 ± 0.06	2.33 ± 0.07	0.04	0.0001	0.001	0.002
SIRI	0.76 ± 0.01	0.83 ± 0.02	0.80 ± 0.02	0.79 ± 0.03	0.89 ± 0.04	0.03	NS	NS	0.02
PT	0.99 ± 0.004	1.06 ± 0.02	1.04 ± 0.02	1.02 ± 0.02	1.13 ± 0.05	0.03	NS	NS	0.0009
CRP (mg/L)	0.65 ± 0.05	1.15 ± 0.14	1.05 ± 0.13	1.67 ± 0.15	1.43 ± 0.27	NS	NS	NS	0.01

Data are presented as means ± standard errors; N, sample size; HTN, hypertension; HLD, hyperlipidemia, T2DM, type 2 diabetes mellitus; CVD, cardiovascular disease; BMI, body mass index; WHR, waist/hip ratio; FM/WT, fat mass/weight ratio; SMM/WT, skeletal muscle mass/weight ratio; TBW, total body water; ECW, extracellular water; GDF-15, growth differentiation factor-15; L/A ratio, leptin/adiponectin ratio; SIRI, systemic inflammation response index; PT, prothrombin time; CRP, C-reactive protein. P1–4 are the significance levels achieved upon comparison of those affected by CVD and the three comorbidities with the control group, determined by a *t*-test; NS, non-significant; *p*-values were obtained after controlling for sex and age.

**Table 2:** Mixed-effects logistic regression analysis exploring the associations between covariates and comorbidities.

Dependent Variable									
	(1) HTN			(2) HLD			(3) T2DM		
Independent Variable	OR (95% CI)	Β (SE)	*p*	OR (95% CI)	Β (SE)	*p*	OR (95% CI)	Β (SE)	*p*
Age	3.40 (2.33–4.96)	1.22 (0.19)	2.12 × 10^−10^	3.11 (2.39–4.04)	1.13 (0.13)	1.15 × 10^−17^	1.29 (0.96–1.49)	0.26 (0.14)	NS
ECW	1.63 (1.28–2.07)	0.49 (0.12)	5.31 × 10^−5^	1.05 (0.86–1.29)	0.05 (0.10)	NS	1.20 (0.96–1.49)	0.18 (0.11)	NS
GDF-15	1.18 (0.91–1.53)	0.17 (0.13)	NS	1.15 (0.89–1.48)	0.14 (0.12)	NS	1.87 (1.44–2.42)	0.62 (0.13)	0.000001
L/A ratio	1.53 (1.19–1.96)	0.42 (0.12)	0.0007	1.12 (0.90–1.40)	0.11 (0.11)	NS	1.35 (1.04–1.75)	0.30 (0.13)	0.02
HTN	-	-	-	3.75 (2.44–5.75)	1.32 (0.21)	1.13 × 10^−8^	1.62 (1.00–2.64)	0.48 (0.24)	0.04
HLD	4.41 (2.48–7.84)	1.48 (0.29)	4.21 × 10^−7^	-	-	-	7.06 (4.12–11.83)	1.95 (0.26)	1.09 × 10^−12^
T2DM	1.89 (1.11–3.21)	0.63 (0.26)	0.01	6.43 (3.86–10.70)	1.86 (0.25)	7.25 × 10^−12^	-	-	-

Data are reported as odds ratios (ORs) with 95% confidence intervals [ORs (95% CIs)], with corresponding Beta and standard errors B (SE) and *p*-values; HTN, hypertension; HLD, hyperlipidemia; T2DM, type 2 diabetes mellitus; ECW, extracellular water; GDF-15, growth differentiation factor-15; L/A ratio, leptin/adiponectin ratio. In the initial stage of the study, the following independent variables were tested in stepwise forward manners: age, sex, BMI, waist circumference, FM/WT, ECW, GDF-15, follistatin, chemerin, L/A ratio, HTN, HLD, and T2DM. Only statistically significant terms are shown in the table. All quantitative variables were standardized before statistical analysis; NS, non-significant.

**Table 3:** Mixed-effects logistic regression analysis exploring the associations between covariates and CVD.

**Dependent Variable: CVD. Stage 1.**			
**Independent Variable**	**OR (95% CI)**	**B (SE)**	***p***
Age	2.67 (1.92–3.72)	0.98 (0.17)	5.84 × 10^−9^
ECW	1.58 (1.25–1.99)	0.46 (0.11)	0.0001
GDF-15	2.42 (1.78–3.30)	0.88 (0.16)	1.96 × 10^−8^
L/A ratio	1.72 (1.33–2.22)	0.54 (0.13)	2.73 × 10^−5^
Lymphocyte	1.50 (1.21–1.85)	0.40 (0.10)	0.0001
SIRI	1.49 (1.19–1.87)	0.40 (0.11)	0.0001
MLR (*χ*^2^) = 241.61, *p* < 0.00001			
**Dependent Variable: CVD. Stage 2.**			
**Independent Variable**	**ROR (95% CI)**	**B (SE)**	***p***
ECW	1.35 (1.06–1.72)	0.30 (0.12)	0.01
GDF-15	1.85 (1.39–2.46)	0.61 (0.14)	2.38 × 10^−5^
Lymphocyte	1.51 (1.21–1.88)	0.41 (0.11)	0.0002
HTN	10.89 (6.46–18.38)	2.38 (0.26)	2.00 × 10^−14^
HLD	2.49 (1.43–4.33)	0.91 (0.28)	0.001
T2DM	1.93 (1.12–3.33)	0.66 (0.27)	0.01
MLR (*χ*^2^) = 330.60, *p* < 0.000001			

Data are reported as odds ratios (ORs) with 95% confidence intervals [ORs (95% CIs)], with corresponding Beta and standard errors B (SE) and *p*-values; ECW, extracellular water, GDF-15, growth differentiation factor-15; L/A ratio, leptin/adiponectin ratio; SIRI, system inflammation response index. In the initial stage of the study, the following independent variables were tested in stepwise forward manners: age, sex, BMI, waist circumference, FM/WT, ECW, GDF-15, follistatin, chemerin, L/A ratio, HTN, HLD, T2DM, SIRI, and lymphocyte count. Only statistically significant terms are shown in the table. All quantitative variables were standardized before statistical analysis. The comparison of the models by the Akaike information criterion (AIC) showed that the second model fits better; AIC Stage_2 = 337 < AIC Stage_1 = 461.

## Data Availability

Data are contained within the article and Supplementary Materials.

## Supplementary Materials

The following supporting information can be downloaded at: https://www.mdpi.com/article/10.3390/biomedicines12051066/s1, Table S1: Baseline characteristics of the study population according to sex; Table S2: Pearson correlations between body composition measurements and plasma levels of soluble markers in the study population by sex; male correlations are shown above the diagonal and female correlations below. All the variables were adjusted for age prior to the analysis.
