# Supplementary material for: Analysis of the Associations of Measurements of Body Composition and Inflammatory Factors with Cardiovascular Disease and Its Comorbidities in a Community-Based Study
Source: Biomedicines. 2024 May 11;12(5):1066. doi: 10.3390/biomedicines12051066 (PMC11117926; doi:10.3390/biomedicines12051066)
Supplement: Supplementary file 1 [file biomedicines-12-01066-s001.zip › biomedicines-2989829-supplementary.pdf]

Table S1. Baseline characteristics of the study population according to sex

**Characteristics**	**Male, N=490**	**Female, N=589**	**P**
Age (years)	42.76±0.62	43.20±0.56	NS
BMI (kg/m^2)	27.53±0.19	28.39±0.23	0.005
Waist circumference (cm)	96.22±0.51	94.04±0.60	0.007
FM/WT (kg/kg)	0.25±0.002	0.36±0.003	0.001
SMM/WT (kg/kg)	0.37±0.002	0.27±0.001	0.001
TBW (L)	45.28±0.26	33.44±0.16	0.001
ECW	0.86±0.007	0.99±0.007	0.001
GDF-15 (pg/ml)	520.47±14.69	460.73±13.10	0.002
Follistatin (pg/ml)	612.26±21.37	619.21±23.16	NS
Chemerin (ng/ml)	88.41±1.24	90.41±1.17	NS
Leptin (ng/ml)	11.43±0.47	33.60±0.92	0.001
Adiponectin (μg/ml)	3.36±0.06	4.48±0.07	0.001
L/A ratio	3.93±0.18	8.76±0.29	0.001
Lymphocytes (×10^9/L)	2.40±0.03	2.09±0.02	0.00001
PT	1.04±0.02	0.98±0.01	0.01
CRP (mg/L)	1.26±0.19	1.19±0.15	NS
SIRI	0.87±0.02	0.70±0.01	1.11×10^{-9}

Data presented as mean, standard errors; N, sample size; BMI, body mass index; FM/WT, fat mass/weight ratio; SMM/WT, skeletal muscle mass/weight ratio; TBW, total body water; ECW, extracellular water; GDF-15, growth differentiation factor-15; L/A ratio, leptin/adiponectin ratio; PT, prothrombin time; CRP, C-reactive protein, SIRI, systemic inflammation response index. P# shows significance levels achieved upon comparing sexes by t-test, NS, non-significant.

Table S2. Pearson correlations between measurements of body composition and plasma levels of soluble markers in the study population by sex; male correlations are shown above the diagonal and female below. All the variables were adjusted for age prior to the analysis.

	**BMI**	**Waist**	**FM/WT**	**SMM/WT**	**ECW**	**GDF-15**	**Chemerin**	**Adiponectin**	**Follistatin**	**Leptin**	**L/A ratio**	**SIRI**
**BMI**		0.87[2]	0.76[2]	-0.77[2]	0.58[2]	0.18[2]	0.34[2]	-0.16[2]	0.06[n]	0.67[2]	0.58[2]	0.03[n]
**Waist**	0.88[2]		0.78[2]	-0.79[2]	0.66[2]	0.25[2]	0.36[2]	-0.12[2]	0.08[n]	0.54[2]	0.64[2]	0.03[n]
**FM/WT**	0.86[2]	0.83[2]		-0.96[2]	0.48[2]	0.25[2]	0.33[2]	-0.07[1]	0.04[n]	0.55[2]	0.66[2]	0.06[n]
**SMM/WT**	-0.80[2]	-0.80[2]	-0.94[2]		-0.42[2]	-0.32[2]	-0.34[2]	0.09[1]	-0.05[n]	-0.52[2]	-0.67[2]	-0.07[n]
**ECW**	0.67[2]	0.65[2]	0.65[2]	-0.49[2]		0.23[2]	0.28[2]	0.04[n]	0.03[n]	0.38[2]	0.33[2]	0.04[n]
**GDF-15**	0.42[2]	0.45[2]	0.39[2]	-0.44[2]	0.37[2]		0.27[2]	-0.03[n]	0.09[n]	0.15[2]	0.17[2]	0.17[2]
**Chemerin**	0.48[2]	0.46[2]	0.44[2]	-0.46[2]	0.33[2]	0.44[2]		-0.06[n]	0.16[2]	0.35[2]	0.38[2]	0.06[n]
**Adiponectin**	-0.15[2]	-0.14[2]	-0.06[n]	0.06[n]	-0.01[n]	0.02[n]	-0.02[n]		-0.01[n]	0.04[n]	-0.42[2]	-0.03[n]
**Follistatin**	0.17[2]	0.18[2]	0.17[2]	-0.19[2]	0.14[2]	0.25[2]	0.30[2]	0.06[n]		-0.00[n]	0.00[n]	0.06[n]
**Leptin**	0.61[2]	0.57[2]	0.63[2]	-0.59[2]	0.42[2]	0.36[2]	0.47[2]	0.01[n]	0.11[1]		0.73[2]	-0.01
**L/A ratio**	0.59[2]	0.57[2]	0.60[2]	-0.59[2]	0.34[2]	0.27[2]	0.41[2]	-0.45[2]	0.09[1]	0.77[2]		-0.00[n]
**SIRI**	0.03[n]	0.01[n]	0.08[n]	-0.04[n]	0.06[n]	0.02[n]	0.03[n]	-0.09[1]	0.03[n]	0.07[n]	0.09[n]	
